# Morphological and molecular characterization of *Heterodera ripae,* a new record cyst nematode in the rhizosphere soil of *Fagopyrum esculentum*

**DOI:** 10.1038/s41598-024-60826-9

**Published:** 2024-04-30

**Authors:** Zaifu Yang, Hui Zhang, Zhaochun Jiang, Yan Wu, Mingrui Liu

**Affiliations:** 1 Department of Plant Pathology, College of Agriculture, Guizhou University, Guiyang, 550025 China; 2 Institute of Vegetable Industry Technology Research, Guizhou University, Guiyang, 550025 China; 3 Guizhou Station of Plant Protection and Quarantine, Guiyang, 550001 China

**Keywords:** Cyst nematode, Buckwheat, Phylogenetic analysis, Ecology, Zoology

## Abstract

Numerous plant parasitic nematodes (PPNs) have the potential to inflict considerable damage on agricultural crops. Through a comprehensive survey aimed at identifying PPNs affecting crops, cyst nematodes were isolated from the rhizosphere soil of buckwheat (*Fagopyrum esculentum*). Employing both molecular and morphological techniques, this cyst nematode was conclusively identified as *Heterodera ripae*. Notably, this represents the first documented occurrence of this particular cyst nematode species within the rhizosphere soil of *F. esculentum*.

## Introduction

Cyst nematodes represent a critical category of plant-parasitic nematodes, with a global distribution that impacts agriculture in multiple countries. These nematodes compromise yields across a variety of essential crops, ranging from cereals and rice to potatoes and soybeans. The genera *Heterodera* and *Globodera* include most of the economically detrimental cyst nematode species.

Originally, Sharma's publication indicated that only 12 species within the *Heterodera* genus were known^1^. However, current taxonomic evaluations recognize 82 nominal species, clustered into nine distinct subgroups—Afenestrata, Avenae, Bifenestra, Cardiolata, Cyperi, Goettingiana, Humuli, Sacchari, and Schachtii—based on morphological traits^2,3^. Recent years have also seen the identification of new species such as *H. dunensis*, *H. microulae* and *H. amaranthusiae*^4–6^. Some *Heterodera* species rank among the world's most damaging plant pests, second only to root-knot nematodes^7^. These nematodes engage in specialized parasitic interactions with their host plants, inducing the formation of a unique feeding structure called the syncytium. Females of the species also exhibit the capability to convert their cuticles into robust capsules for protecting their eggs^1^.

In a 2022 study, Peng et al.^8^ shed light on the adverse impacts of *Globodera rostochiensis* in Hezhang and Weining counties of Guizhou Province. This nematode is particularly devastating to potatoes and causing considerable damage to the plant's subterranean components and significant yield losses. On average, *G. rostochiensis* reduces potato yields by 30%, and in severe cases, losses can escalate to between 80 and 90%^9^.

Given the significant cultivation of potatoes in Guizhou, spanning over an area of more than 70,000 hectares every year, we conducted a survey to explore the prevalence of cyst nematodes in the region. During this investigation, we discovered *Heterodera ripae.*

Previously, *H. ripae* had been discovered parasitizing the roots of common nettle (*Urtica dioica*) in countries such as Russia, Germany, and Belgium, dating back to 1982^10^. Over time, its presence has been noted in various other countries, including Estonia, Latvia, Armenia, Moldova, Ukraine, Bulgaria, Germany, Belgium, Slovakia, Greece, Sweden, Spain, and China^11–15^. Until recently, *H. ripae* was predominantly found in the rhizosphere soil or roots of *Urtica* spp. However, during a crop cyst nematode survey in Liupanshui, Guizhou Province, *H. ripae* was isolated from the rhizosphere soil of *Fagopyrum esculentum* in 2020. This marks the first known occurrence of *H. ripae* in the rhizosphere soil of a crop other than *Urtica* spp.

## Materials and methods

### Collection of soil sample

The soil samples were collected from the rhizosphere of *F. esculentum* in Wumeng Town, Panzhou County, Liupanshui City, Guizhou Province. By employing a random sampling technique, soil samples ranging from 5 to 10 cm^3^ were extracted. A total of fifteen samples were collected and subsequently combined into a composite sample weighing approximately 3 kg. This composite sample was then placed in disposable plastic bags for further analysis. Pertinent details regarding the soil samples, such as location, depth, and date of collection, were meticulously recorded for future reference.

### Nematode extraction

Each collected soil sample was rigorously mixed to achieve a homogeneous composition. Subsequently, a 100 g subsample was air-dried at a temperature of 37 °C over a period of two days to prepare it for cyst extraction, as outlined in previous studies^16,17^.

The cysts were isolated using the simple floating method. 200 g of the mixed soil was weighed and transferred into a 2000 mL triangular bottle, followed by the addition of 1800 mL of water. After 5–10 min, the floating objects were filtered through a 250 μm sieve. The cysts were then carefully selected and collected on this sieve. Second-stage juveniles were directly extracted from these cysts by cutting. Post-extraction, the cysts were carefully gathered using forceps and transferred to a watch glass filled with tap water. This procedure was conducted under the stereomicroscope (S9i Leica, Germany) with 20 ×. The collected cysts were then stored at a temperature of 4 °C for subsequent analyses.

### Morphological and morphometric for cysts and J2s

A total of ten cysts were selected for comprehensive morphological measurement. Both the cysts and second-stage juveniles (J2s) were closely examined under a Zeiss Axioscope 5 microscope (Germany) as well as a Keyence VHX-7000 digital stereoscopic microscope (Japan). Photographic documentation was carried out to capture their distinct features. Morphological identification was performed in accordance with the taxonomic descriptions provided by Willmott et al. and Subbotin et al.^18,19^.

For slide preparation focusing on the vulval cone and second-stage juveniles, established methodologies were followed. Slides of the vulval cone were prepared and photographed using the techniques outlined by Li et al.^4^. Preparation of second-stage juveniles was conducted based on methods described by Zhuo et al.^20^. Specifically, the test tube containing the suspension of second-stage juveniles was heated in a water bath at 65 °C for a duration of 2 min, and subsequently fixed using a formalin-glacial acetic acid (FA) solution.

Utilizing water as the floating medium, the fixed nematodes were carefully transferred onto a cover glass. Thereafter, they were observed, measured, and photographed under a light microscope for detailed analysis.

### Genomic DNA extraction

Genomic DNA extraction from the cyst nematode was carried out using the protocol developed by Reid and Pickup^17^. A singular cyst was chosen for the procedure, and a small quantity of eggs along with second-stage juveniles were aspirated following puncture of the cyst. These materials were then placed into a sterile 200 μL PCR tube. Subsequently, 20 μL of deionized water (ddH_2_O) and 2 μL of 10 × PCR buffer containing Mg^2+^ were added to the tube. The sample was stored in a − 80 °C refrigerator overnight for stabilization. Upon thawing, 2 μL of protease K (concentration: 20 mg/mL) was added to the sample, which was then incubated at 65 °C for 90 min to facilitate cellular lysis. This was followed by a further incubation at 95 °C for 30 min to inactivate the protease K enzyme. The tube was then centrifuged at a speed of 14,000 rpm for a duration of 3 min to sediment any particulate matter.

The extracted genomic DNA was subsequently stored at − 20 °C, making it available for future polymerase chain reaction (PCR) analyses.

### PCR amplification

In this study, the following primer sets were employed for DNA amplification: TW81 (5′-GTTTCCGTAGGTGAACCTGC-3′) and AB28 (5′-ATATGCTTAAGTTCAGCGGGT-3′) for amplifying the rRNA internal transcribed spacer region (ITS)^21^; D2A (5′-ACAAGTACCGTGAGGGAAAGTTG-3′) and D3B (5′-TCGGAAGGAACCAGCTACTA-3′) for amplifying the D2–D3 expansion segments of the 28S rRNA gene^22^; Het-coxiF (5'-TAGTTGATCGTAATTTTAATGG-3′) and Het-coxiR (5′-CCTAAAACATAATGAAAATGWGC-3′) for amplifying the partial mitochondrial cytochrome oxidase subunit I (mt*COI*) gene^23^.

Polymerase chain reactions (PCRs) were executed in 20 µL reaction mixtures that included 10 µL of 2 × Bench Top Taq Master Mix (Biomiga, AT1201, San Diego), 7 µL of ddH_2_O, 1 µL of each the forward and reverse primers (at a concentration of 10 µM), and 1 µL of the genomic DNA template.

The amplification protocols for the ITS and D2–D3 regions were as follows: an initial pre-denaturation at 94 °C for 5 min, followed by 35 cycles of denaturation at 94 °C for 50 s, annealing at 55 °C for 50 s, and extension at 72 °C for 90 s. A final extension step at 72 °C for 7 min was included^24^.

For the *COI* gene, the PCR amplification consisted of an initial 4 min at 94 °C, followed by 40 cycles of 1 min at 94 °C, 1 min at 45 °C, and 1 min 30 s at 72 °C. A final extension step was carried out at 72 °C for 10 min^22^.

Post-amplification, the PCR products were subjected to electrophoretic separation on a 1% agarose gel using 1 × TAE buffer, and run for a duration of 30 min. Gel visualization was accomplished using a UV transilluminator.

### Sequencing and phylogenetic analysis

Following the amplification process, the PCR products were purified using the DiaSpin PCR Product Purification Kit (Sangon Biotech (Shanghai) Co., Ltd.), adhering to the guidelines provided in the manufacturer's manual. The purified DNA samples were then submitted to SinoGenoMax Corporation in Beijing, China, for sequencing analysis.

The resulting sequence data were edited using the ContigExpress application to eliminate low-quality bases. These edited sequences were uploaded to the National Center for Biotechnology Information (NCBI), and obtained similarity matches with existing *Heterodera* species whose nucleotide sequences are already archived in the NCBI database. The GenBank accession numbers used in the phylogenetic analysis are shown in Table 1. Outgroup taxa for each dataset were identified based on prior research. For the ITS and D2–D3 datasets, *Meloidodera sikhotealinensis* and *Cryphodera brinkmani* were used as outgroups, respectively, while *Rotylenchus eximius* and *R. urmiaensis* served as outgroups for the *COI* gene dataset.

**Table 1:** GenBank accession numbers used in the phylogenetic analysis.

Species	ITS Acc. Nos	species	D2D3 Acc. Nos	species	mt*COI* Acc. Nos	species	mt*COI* Acc. Nos
*Heterodera ripae*	AY347927.1	*H. ripae*	OQ064081.1	*H. mani*	MG523095.1	*H. ripae*	MT808356.1
*H. ripae*	KM114205.1	*H. ripae*	OQ064080.1	*H. mani*	KU147203.1	*H. ripae*	OQ048424.1
*H. ripae*	DQ846902.1	*H. ripae* (LPS)	OR468129.1	*H. mani*	MG523097.1	*H. ripae*	MT808354.1
*H. ripae*	AF274407.1	*H. litoralis*	DQ328691.1	*H. australis*	KU147202.1	*H. ripae*	MT808355.1
*H. ripae*	OQ048459.1	*H. fici*	LT996914.1	*H. sturhani*	KU147199.1	*H. ripae*	MW279112.1
*H. ripae*	AF393840.1	*H. salixophila*	DQ328690.1	*H. pratensis*	KU147200.1	*H. ripae*	ON007078.1
*H. ripae* (LPS)	OR468061.1	*H. salixophila*	MT210880.1	*H. avenae*	MG523007.1	*H. ripae*	ON007079.1
*H. humuli*	AY347926.1	*H. trifolii*	GU475089.1	*H. arenaria*	MG522942.1	*H. ripae*	MW279111.1
*H. humuli*	AF498384.1	*H. glycines*	GU475087.1	*H. arenaria*	MG522944.1	*H. ripae*	MW279110.1
*H. humuli*	AF274408.1	*H. schachtii*	GU475088.1	*H. aucklandica*	MG523089.1	*H. ripae*	MT808353.1
*H. vallicola*	AF393841.1	*H. latipons*	DQ328687.1	*H. ustinovi*	MG523094.1	*H. ripae*	MT808352.1
*H. fici*	AF498385.1	*H. hordecalis*	LT159829.1	*H. atipons*	MG523129.1	*H. ripae*	MT808351.1
*H. litoralis*	AF274410.1	*H. avenae*	HM560821.1	*H. sojae*	MK188447.1	*H. ripae*	MT808347.1
*H. turcomanica*	AF498386.1	*H. filipjevi*	MG859980.1	*H. guangdongensis*	MF425735.1	*H. ripae*	MT808346.1
*H. salixophila*	AF274406.1	*H. sorghi*	DQ328689.1	*H. cyperi*	MG857126.1	*H. ripae*	MT808345.1
*H. salixophila*	AF274405.1	*H. oryzicola*	DQ328694.1	*H. mothi*	MH144208.1	*H. ripae*	MT808344.1
*H. salixophila*	MN480559.1	*H. elachista*	MN699477.1	*H. elachista*	MH144207.1	*H. ripae*	MT808343.1
*H. schachtii*	AF498389.1	*H. goettingiana*	MH032764.1	*H. elachista*	KC618472.1	*H. ripae*	MT808342.1
*H. glycines*	AF498387.1	*H. cruciferae*	KP114546.1	*H. elachista*	KC618473.1	*H. ripae*	MT808341.1
*H. ciceri*	AF274393.1	*H. carotae*	MN818689.1	*H. medicaginis*	MK093178.1	*H. ripae*	MT808340.1
*H. trifolii*	AF498388.1	*H. urticae*	DQ328696.1	*H. medicaginis*	MK093179.1	*H. ripae*	MT808339.1
*H. sacchari*	AF274403.1	*H. cynodontis*	DQ328698.1	*H. glycines*	KC172914.1	*H. ripae*	MT808338.1
*H. sorghi*	AF274404.1	*Meloidodera sikhotealiniensis*	DQ328706.1	*H. schachtii*	LC208708.1	*H. ripae*	MT808348.1
*H. latipons*	AF498382.1	*Cryphodera brinkmani*	JQ965677.1	*H. schachtii*	KC172918.1	*H. ripae*	MT808349.1
*H. hordecalis*	AF498381.1	–	–	*H. cicer*	KC172919.1	*H. ripae*	MT808350.1
*H. filipjevi*	GU565574.1	–	–	*H. trifolii*	MG523138.1	*H. koreana*	LC202193.1
*H. avenae*	AF498378.1	–	–	*H. betae*	LC208706.1	*H. goettingiana*	KY129829.1
*H. ustinovi*	AF274400.1	–	–	*H. betae*	LC208707.1	*H. goettingiana*	KY129831.1
*H. bifenestra*	AF274384.1	–	–	*H. trifolii*	MG682349.1	*H. carotae*	MG563235.1
*H. cynodontis*	AF274386.1	–	–	*H. daverti*	KC172915.1	*H. cruciferae*	MG563234.1
*H. cyperi*	AF274388.1	–	–	*H. hordecalis*	MG523146.1	*H. pratensis*	KC172916.1
*H. cyperi*	AF498392.1	–	–	*H. filipjevi*	KC172911.1	*H. carotae*	MG563229.1
*H. oryzicola*	AF274387.1	–	–	*H. filipjevi*	MG523083.1	*H. oryzae*	MT823012.1
*H. elachista*	AF498391.1	–	–	*H. filipjevi*	MG523084.1	*Rotylenchus urmiaensis*	KP718972.1
*H. goettingiana*	AF498374.1	–	–	*H. humuli*	MW279120.1	–	–
*H. carotae*	AF274413.1	–	–	*H. humuli*	MT808368.1	–	–
*H. cruciferae*	AF274411.1	–	–	*H. humuli*	MW279121.1	–	–
*H. urticae*	AF274412.1	–	–	*H. humuli*	MW279122.1	–	–
*Meloidodera sikhotealiniensis*	AF274419.1	–	–	*H. vallicola*	MT808357.1	–	–
*Cryphodera brinkmani*	AF274418.1	–	–	*H. ripae* (LPS)	OR468243.1	-	-

Sequence alignment for the ITS, D2–D3, and *COI* genes was performed using the online version of MAFFT v. 7 (https://mafft.cbrc.jp/alignment/server/), and the alignments were manually refined for accuracy. Subsequently, a phylogenetic tree was constructed using MEGA 6.06 software. The Neighbor-Joining (NJ) method was employed for tree construction, and the tree's reliability was assessed through Bootstrap analysis, involving 1000 replicates. This comprehensive approach culminated in the generation of the phylogenetic tree, serving as a vital tool for evaluating and analyzing the genetic relationships among the studied nematode species.

### Declarations

All the experimental research and field studies on plants, as well as the collection of plant material in this manuscript, comply with relevant institutional, national, and international guidelines and legislation.

## Results

### Morphological description

**Figure 1:**
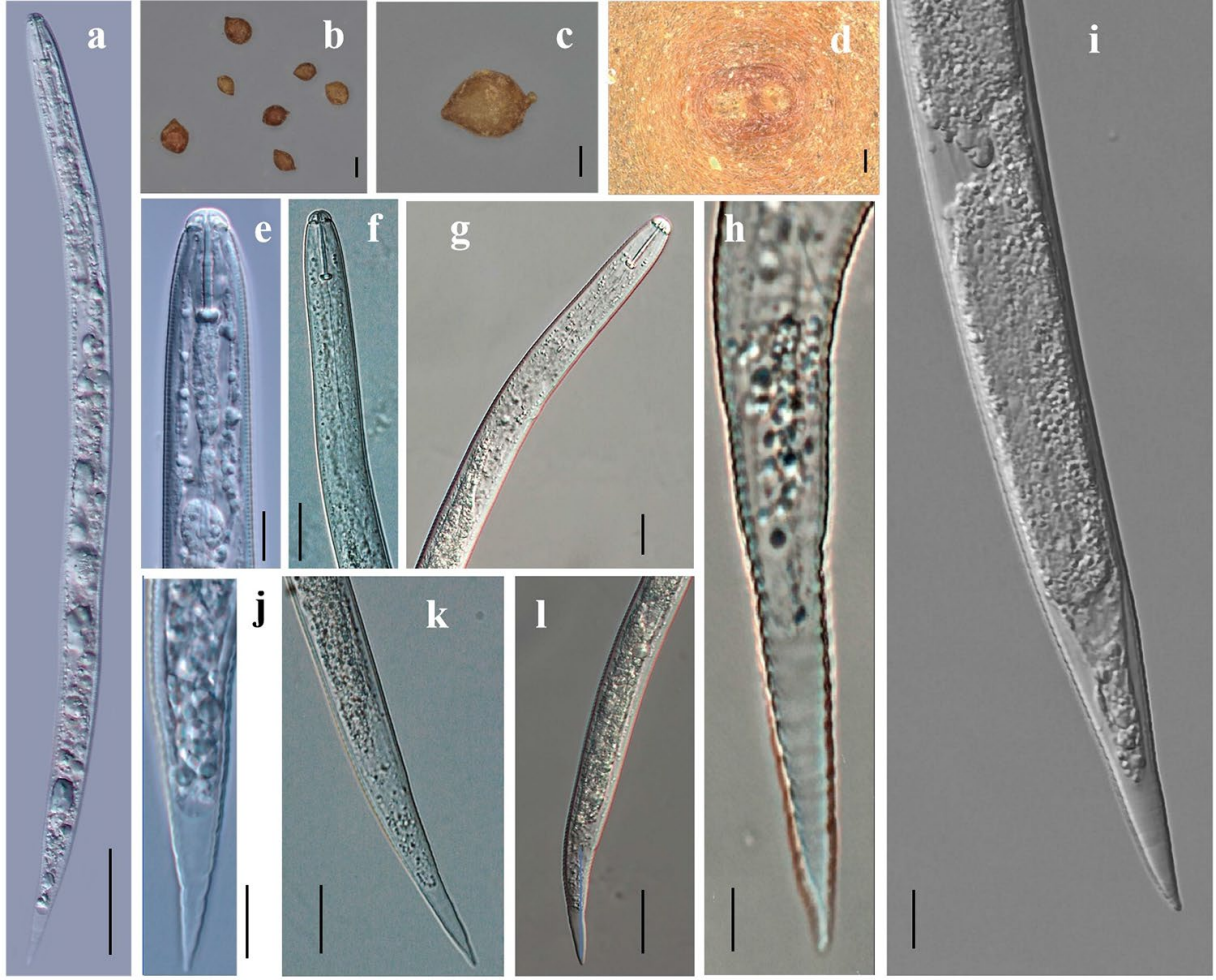
Morphological characteristics of cyst, vulval cone, and second-stage juveniles (J2s) of *H.ripae*. (**a**) Entire J2 body, (**b**) Cysts, (**c**) Lemon-shaped cyst, (**d**) Vulva, (**e**)–(**g**) Anterior end of J2s, (**h**)–(**l**) Posterior end of J2s. Scale bars: (a) = 50 μm, b = 100 μm, c = 100 μm, d = 10 μm, e = 10 μm, (f, g, k, l)=20 μm; (h, i)=5 μm, j = 10 μm.

Cyst morphology (Fig. 1b–d). The cysts were lemon-shaped, featuring a distinct and relatively wide vulval cone. Their color ranged from yellow to pale brown and darkened as they aged. The surface was adorned with ridges arranged in an irregular zigzag pattern. A pronounced neck was often visible, frequently forming an angle against the body axis. The vulval cone was bifenestrate, and bullae were absent, with a weak underbridge (Fig. 1d). Morphometrics of cysts (Table 2). The body length (excluding the neck) varied between 388 and 473 µm, with an average length of 439 µm, while the body width ranged from 284 to 416 µm, with an average of 344 µm. The fenestrate length ranged from 35 to 53 µm, with an average of 44. The vulval bridge width varied between 6.5 and 12 µm, with an average of 9.1 µm. The underbridge lenth ranged from 52 to 95 µm, with an average of 69.9 µm.

**Table 2:** Morphometrics of cysts and second-stage juveniles (J2) of different populations of *Heterodera ripae.*

Population	*H. ripae* *Fagopyrum esculentum* Liupanshui China	*H. ripae* *Urtica dioica* Moscow Russia^10^	*H. ripae* *Urtica dioica* Münster Germany^10^	*H. ripae* *Urtica* sp. Shenyang China^14^	*H. ripae* *Urtica dioica* León and Burgos Spain^13^
Cysts					
n	10	50	25	10	25
Length (excluding neck)	439 ± 31.9 (388–473)	462 ± 7.9 (348–580)	415 ± 9.6 (289–497)	437.6 ± 37.4 (379.4–488.4)	418 (295–489)
Width	344 ± 38.9 (284–416)	327 ± 9.0 (212–454)	307 ± 9.4 (214–383)	321.7 ± 40.3 (262.0–378.6)	310 (208–375)
a (length/width)	1.3 ± 0.1 (1.1–1.5)	1.4 ± 0.3 (1.1–1.8)	1.4 ± 0.02 (1.1–1.7)	1.4 ± 0.1 (1.3–1.5)	
Vulval areas (n)	10	35	15	10	25
Fenestrate length	44 ± 7.1 (35–53)	46 ± 0.8 (31–58)	46 ± 1.5 (38–56)	47.6 ± 3.6 (42.9–53.7)	46.5 (39–58)
Fenestral width	–	–	–	–	25.1 (25.2–30.9)
Mean semifenestral width	23.8 ± 3.9 (20–28)	26.3 ± 0.7 (16.6–38.2)	24.8 ± 0.9 (18–31.7)	25.3 ± 2.4 (22.1–29.5)	–
Vulval slit length	31.9 ± 4.3 (28–39)	33.8 ± 0.6 (28.2–41.5)	33 ± 0.9 (26–41)	34.2 ± 2.3 (31.2–37.6)	33 (26–40)
Vulval bridge width	9.1 ± 1.7 (6.5–12)	10.7 ± 0.9 (6.6–18.2)	9 ± 0.5 (6–14)	10.0 ± 1.0 (9.0–11.9)	–
Underbridge lenth	69.9 ± 13.6 (52–95)	78 ± 2.3 (70–88)	69 ± 2.2 (50–90)	75.1 ± 3.3 (70.2–79.7)	69.2 (51–90)
Vulva-anal distance	–	47 ± 1.4 (36–63)	43 ± 3.9 (24–75)	47.1 ± 5.8 (39.4–55.2)	-
J2					
N	10	52	25	12	20
L (body length)	369 ± 21.2 (340–415)	373 ± 2.1 (342–407)	359 ± 2.3 (336–381)	400.9 ± 30.1 (352.8–444.9)	359.3 (338–380)
a (L/width)	20.2 ± 1.7 (17.9–24.5)	20.7 ± 0.1 (18.7–22.8)	21.6 ± 0.1 (19.7–22.6)	19.5 ± 1.9 (16.7–23.9)	–
b	3.4 ± 0.3 (3–3.8)	3.3 ± 0.03 (2.9–4.0)	3.7 ± 0.04 (3.5–4.0)	3.5 ± 0.3 (2.9–3.9)	–
c	6.5 ± 0.3 (6–7.1)	8.0 ± 0.1 (7.4–10.0)	8.1 ± 0.1 (7.1–8.9)	9.3 ± 1.6 (7.2–12.2)	–
c’	4.8 ± 0.2 (4.4–5.2)	4.1 ± 0.04 (3.4–4.5)	4.2 ± 0.1 (3.8–5.0)	3.6 ± 0.2 (3.2–4.0)	–
Stylet length	20.3 ± 0.5 (20–21)	21.7 ± 0.1 (20.3–23.5)	22.1 ± 0.1 (20.8–23.8)	21.6 ± 0.7 (20.2–22.4)	22.1 (21–24)
Body with at:					
Mid-body	18.3 ± 0.8 (17–19)	18.0 ± 0.1 (16.3–20.1)	16.7 ± 0.1 (15.2–18.4)	20.7 ± 2.0 (18.1–24.2)	–
Anus	12 ± 0.8 (11–13)	11.2 ± 0.1 (9.9–12.8)	10.7 ± 0.1 (10.0–11.6)	-	–
Hyaline part of tail length (H)	25 ± 1 (23.0–26.0)	22.9 ± 0.3 (18.1–27.5)	23 ± 0.3 (18.4–25.6)	21.6 ± 0.7 (20.2–22.4)	23.3 (18–25)
Tail length	57 ± 4 (48–61)	47 ± 0.4 (36–50)	45 ± 0.6 (41–52)	43.7 ± 6.1 (33.6–52.2)	45.6 (41–52.5)
H/stylet length	1.2 ± 0.05 (1.2–1.3)	1.1 ± 0.02 (0.8–1.3)	1.0 ± 0.01 (0.8–1.2)	1.0 ± 0.1 (0.9–1.2)	–

All measurements are in μm and in the form: mean ± s.d. (range).

The J2s exhibited a slightly ventrally curved body (Fig. 1a). Their stylets were robust, featuring rather wide and slightly anteriorly projecting knobs (Fig. 1e). The median bulb was oval-shaped, and pharyngeal glands were well developed (Fig. 1e–g). The excretory pore was positioned just anterior to the level of the pharyngo-intestinal junction and immediately posterior to the hemizonid. The tail was conical, terminating in a finely rounded end (Fig. 1h–l). Morphometrics of J2s (Table 2). Measurements indicated a body length that ranged from 340 to 415 µm (mean of 369 µm); a stylet length between 20 and 21 µm (mean of 20 µm); and a hyaline part of the tail that ranged from 23 to 26 µm (mean of 25 µm). The lateral field exhibited four lines. No males were observed in the samples. The morphological data were consistent with previous records of *H. ripae*^10,11,13,14^.

### Phylogenetic analysis

**Figure 2:**
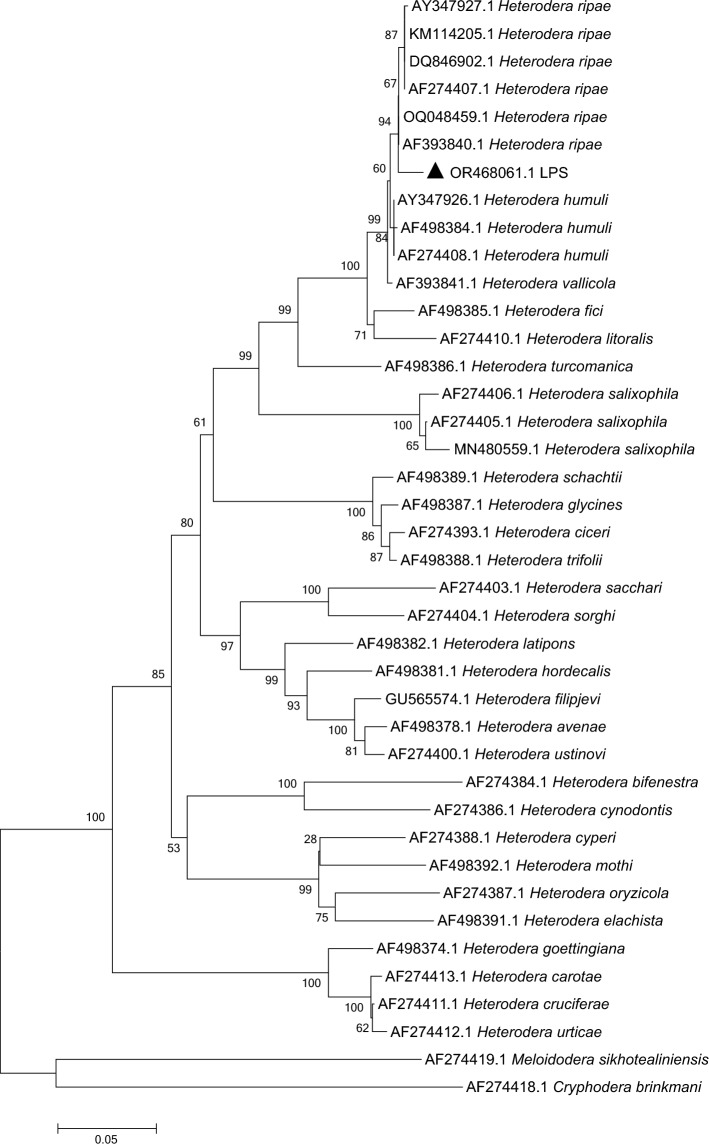
Phylogenetic tree constructed based on the ITS sequences of the genus *Heterodera*. Note: Triangles represent the population from Liupanshui. The total nucleic acid positions was 966. Using the Neighbor-joining statistical method, the Maximum Composite Likelihood model constructed the phylogenetic tree, which was detected by 1000 Bootstrap replications. Bootstrap values are given for the appropriate clades.

**Figure 3:**
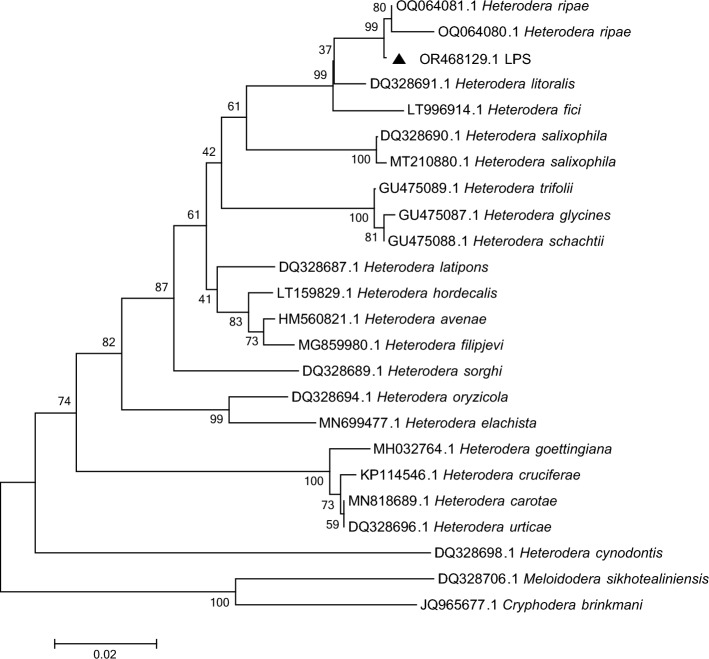
Phylogenetic tree constructed based on the D2–D3 gene sequences of the genus *Heterodera*. Note: Triangles represent the population from Liupanshui. The total nucleic acid positions was 761. Using the Neighbor-joining statistical method, the Maximum Composite Likelihood model constructed the phylogenetic tree, which was detected by 1000 Bootstrap replications. Bootstrap values are given for the appropriate clades.

**Figure 4:**
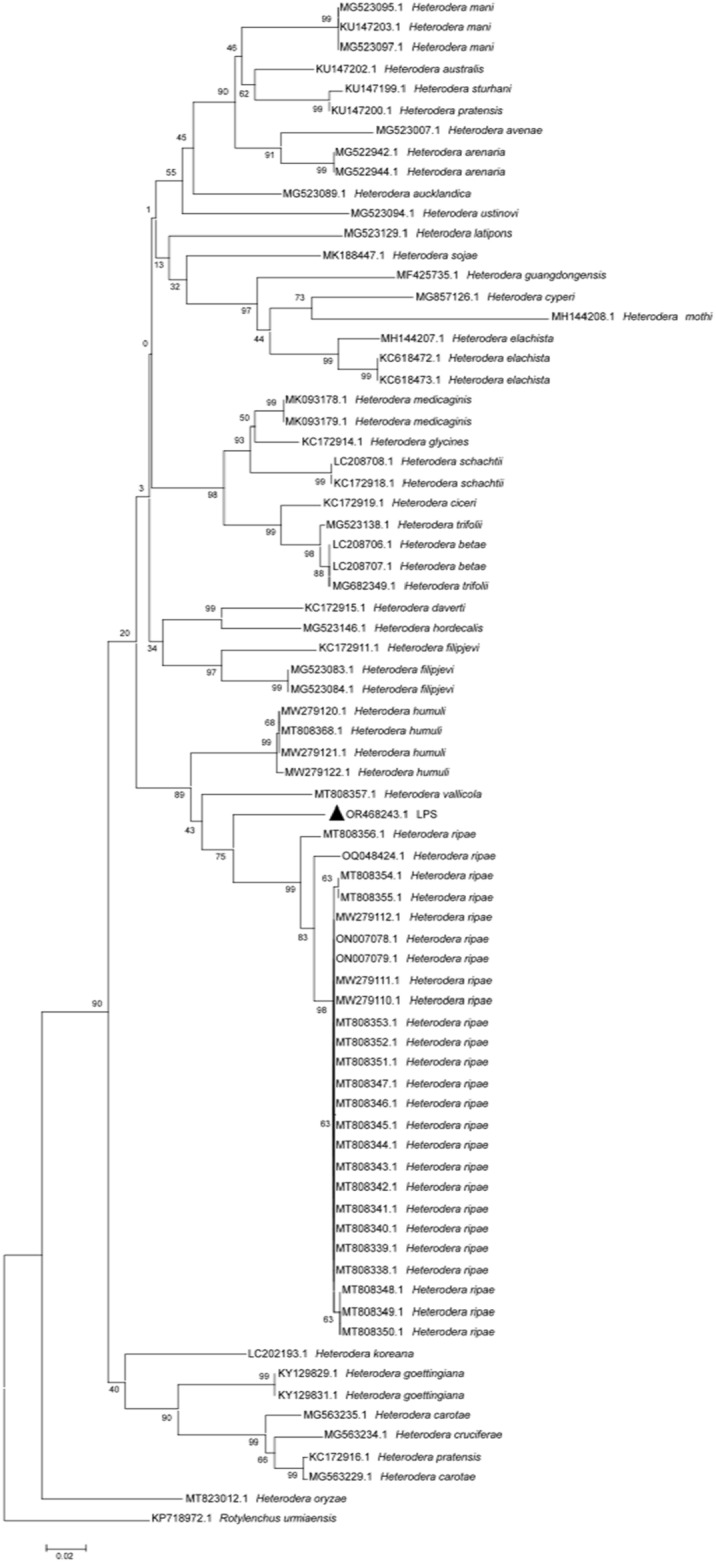
Phylogenetic tree constructed based on the mt*COI* gene sequences of the genus *Heterodera*. Note: Triangles represent the population from Liupanshui. The total nucleic acid positions was 389. Using the Neighbor-joining statistical method, the Maximum Composite Likelihood model constructed the phylogenetic tree, which was detected by 1000 Bootstrap replications. Bootstrap values are given for the appropriate clades.

Sequencing results for the nematode population (LPS) included the ITS (OR468061.1), 28S rRNA D2–D3 (OR468129.1), and *COI* gene (OR468243.1) were upload to NCBI database. Phylogenetic trees were constructed with sequences from the genus of *Heterodera.* The phylogenetic tree of the ITS gene (Fig. 2) represented that LPS (OR468061.1) clustered in the same branch with *H. ripae* (AY347927.1, DQ846902.1, AF274407.1, KM114205.1, OQ048459.1, AF393840.1). LPS (OR468129.1) gathered with *H. ripae* (OQ064081.1, OQ064080.1) in one branch in the phylogenetic tree of 28S rRNA D2–D3 (Fig. 3). LPS (OR468243.1) clustered with all current *COI* gene sequences (NCBI database) of *H.ripae* in the same branch (Fig. 4).

Phylogenetic analysis corroborated these morphological observations, the LPS cyst nematode population was identified as *H. ripae*.

## Discussion

The nematode population under study can be conclusively identified as *H. ripae* based on both morphological and genetic evidence. The results of this study extend understanding of the distribution and host range of *H. ripae*. While our findings align closely with previous studies on the species, they also present new, important observations. Our study confirms that *H. ripae* is closely related to *H. humuli*, with the key differentiating factor being the average length of the J2 tail, specifically the hyaline tail length^10,11,13,14^. This distinction is consistent with existing literature, further solidifying our understanding of the taxonomy and identification characteristics of the Humuli group of cyst nematodes.

While the morphological characteristics generally align with previous reports, noted slight variations in specific measurements. The body length range of J2 and the hyaline part of the tail in our population are slightly broader compared to earlier studies. These variations could be attributed to environmental factors, geographical distribution, or even a slight evolution in the species, warranting further investigation.

Most significantly, our study provides the first evidence of *H. ripae* in the rhizosphere soil of buckwheat (*F. esculentum*), a previously unreported host for this nematode species. Interestingly, although the host plants of *H. ripae* are traditionally understood to be *Urtica* spp.^10^. Our collection sites did not indicate the presence of these plants. This finding could have implications for the control and management of *H. ripae* and suggests a need for further research on its host range and geographical distribution. This study also expands our understanding of the geographic distribution of *H. ripae*, marking its presence in the Liupanshui City region.

Our study enriches the existing literature by confirming certain known characteristics of *H. ripae*, introducing new morphometric data, identifying a new potential host plant, and extending its known geographical distribution. These findings could prove useful for future research and for the management of cyst nematodes in agriculture.

## Data Availability

Data is provided within the manuscript.
